# Secreted glucose regulated protein78 ameliorates DSS-induced mouse colitis

**DOI:** 10.3389/fimmu.2023.986175

**Published:** 2023-01-26

**Authors:** Liang Zhao, Yibing Lv, Xiaoqi Zhou, Zilong Guo, Heli Li, Yanyan Guo, Tao Liu, Lei Tu, Liangru Zhu, Juan Tao, Guanxin Shen, Yong He, Ping Lei

**Affiliations:** ^1^ Department of Immunology, School of Basic Medicine, Tongji Medical College, Huazhong University of Science and Technology, Wuhan, China; ^2^ Department of Nuclear Medicine and PET Center, Zhongnan Hospital of Wuhan University, Wuhan, China; ^3^ Department of Dermatology, Affiliated Union Hospital, Tongji Medical College, Huazhong University of Science and Technology, Wuhan, China; ^4^ Department of Gastroenterology, Affiliated Union Hospital, Tongji Medical College, Huazhong University of Science and Technology, Wuhan, China; ^5^ Department of Cancer Center, Affiliated Union Hospital, Tongji Medical College, Huazhong University of Science and Technology, Wuhan, China

**Keywords:** sGRP78, ulcerative colitis, macrophage, DSS, immunomodulatory

## Abstract

The secreted form of 78-kDa glucose-regulated protein (sGRP78) has been widely reported for its property in aiding resolution of inflammatory. However, little is known on its potential in the treatment of colitis. To investigate the expression pattern and functional outcome of GRP78 in ulcerative colitis, its expression was measured in human and murine colitis samples. It was found that GRP78 was spontaneously secreted to a high level in gut, which is a physiological site of immune tolerance. During the active phase of DSS-induced colitis, the sGRP78 level was significantly reduced but rebounded quickly during resolving phase, making it a potential candidate for the treatment of colitis. In the following experiments, the administration of sGRP78 was proved to decrease susceptibility to experimental colitis, as indicated by an overall improvement of intestinal symptoms, restoration of TJ integrity, decreased infiltration of immune cells and impaired production of inflammatory cytokines. And specific cleavage of endogenous sGRP78 could aggravate DSS colitis. Adoptive transfer of sGRP78-conditioned BMDMs reduced inflammation in the gut. We linked sGRP78 treatment with altered macrophage biology and skewed macrophage polarization by inhibiting the TLR4-dependent MAP-kinases and NF-κB pathways. Based on these studies, as a naturally occurring immunomodulatory molecule, sGRP78 might be an attractive novel therapeutic agent for acute intestinal inflammation.

## 1 Introduction

Inflammatory bowel disease (IBD), refers to a group of multifactorial, immunologically-mediated chronic inflammatory diseases, of which Crohn’s disease (CD) and ulcerative colitis (UC) represent the two major forms of disease. IBD can be debilitating and may lead to life-threatening complications, and its incidence and prevalence are increasing worldwide (1). Disturbed intestinal immune system and microbiota, poorly defined environmental triggers, mental health problems, are widely recognized to contribute to the pathogenesis of IBD (2, 3). These contributors are characterized by exposing intestinal cells to various stress conditions, including pro-inflammatory cytokines, neuropeptides, acids, oxidants, toxins or microbiota-derived metabolites (4, 5), causing cellular damage. In response to stresses, intestinal cells mobilize endogenous cytoprotective defense mechanisms to confront injury. Heat shock proteins (HSPs) are stress-responsive molecules involved in pathophysiological process of IBD, and can be induced to a high level by many physiological and environmental stress factors in intestinal cells (6). However, their expression patterns vary depending on intestinal cell types, stress conditions, disease progression, and more, with their functional outcomes contributing to IBD in different aspects (7–9). Given that induction and/or targeted inhibition of specific HSPs have been suggested to ameliorate the condition of disease (7), understanding the expression and function of intestinal HSPs may provide a better guideline for IBD treatment.

As a member of highly conserved HSP70 family, glucose - regulated protein 78 (GRP78, also referred to as BiP), acts as a central regulator of endoplasmic reticulum (ER) homeostasis by playing key roles in nascent protein chain folding, transport and quality control (10). This ER-resident chaperone is upregulated under stress conditions, including hypoxia, inflammatory cytokines, low-calcium, environmental or genetic disturbances and others, to afford cytoprotection and restrict disease progression. Its upregulation is widely used as a sentinel marker under those pathologic conditions (10, 11). Our previous work confirmed that GRP78 overexpression can protect insulinoma NIT-1 cells from cytotoxic T-cell-mediated lysis (12) and enhance survival of CHO cells in response to oxidative stresses (13). Upregulation of GRP78 leads to its ER escape and translocation to the cytosol, nucleus, or the plasma membrane and extracellular fluids (14–16). Secreted GRP78 acts as a resolution-associated molecular pattern (RAMP) to favor the resolution of inflammation (17) by generation of regulatory T-cell (18) and B-cell (19) population, by affecting maturation of dendritic cells (20, 21) and by targeting CD14 to impair production of LPS-induced proinflammatory cytokines (22). sGRP78 helps resolve inflammation in NOD mice (23) and contributes to tumor growth and metastasis by promoting immune tolerance to remodel the tumor microenvironment (24). Hence, both the cytoprotective property of intracellular GRP78 and the immune modulatory property of sGRP78 provide a direction towards determining GRP78’ ability in IBD.

The present study explored the expression pattern and functional outcome of intestinal GRP78 in IBD. It was found that sGRP78 was spontaneously produced to a high level in gut, the important site of immune tolerance, contributing to the maintenance of gut immune homeostasis. In mice, sGRP78 expression was negatively regulated as colitis progresses. Mice administered with sGRP78 displayed mild DSS-induced colitis compared with wild-type littermates and skewed macrophage polarization. Thus, we suggest sGRP78 as a promising novel agent for the treatment of IBD.

## 2 Materials and methods

### 2.1 Animal studies and colitis induction

Female C57BL/6 mice (HFK Bioscience, Beijing, China) were randomly divided into following groups: non-colitic control group; colitic group receiving saline (200 μl); colitic group receiving sGRP78 (10 mg/kg) or selective GRP78 serine proteinase subA (20 μg/mouse) intraperitoneally. Acute colitis was induced in mice with 2.5% DSS (MP Biomedicals, Solon, USA) for five consecutive days, followed by a tap water period until end of experiments. For BMDMs treatment experiments, the day that colitis was firstly induced by DSS was identified as day 0. On day -1 and 1, female C57BL/6 mice (HFK Bioscience, Beijing, China) were injected intraperitoneally with 1×10^6^ sGRP78-induced BMDMs that were treated with recombinant mouse GRP78 for 48 h *in vitro.* None-treated BMDMs were injected intraperitoneally for another group mice as comparison. After mice were sacrificed, colons were isolated to measure their length from cecum to anus and to perform macroscopic, histochemical and biochemical analyses. All animal protocols were approved by the Ethics Committee of Tongji Medical College of Huazhong University of Science and Technology (HUST, Approval ID: S1161/S2534).

### 2.2 Disease activity index (DAI) in mice

The DAI was scored to characterize experimental colitis induction and progression (25, 26). Details are described in the online **Supplementary Section**.

### 2.3 Preparation of blood samples from mice

Before being sacrificed, mice were deeply anaesthetized and the blood was collected from supra orbital sinus. To determine sGRP78, cytokines, plasma was isolated from the blood, immediately frozen, and stored at −80°C until the assays.

### 2.4 Human and mice tissues

Actively inflamed human colonic specimens were obtained from eight UC patients receiving colonoscopy and six non-colitic patients receiving bowel resection for localized colon cancer at Wuhan Union hospital. Colitic mucosa was dissected from inflamed areas. Non-colitic control mucosa was dissected from the specimen near the resection margin of cancer patients which should be at least 5 cm between the tumor and the colonic section line (27–29). Mucosa punches of defined surface area (area = π × r^2^) were prepared using a 4 mm dermal punch biopsy instrument (30). All patients have signed an informed consent. Human studies were approved by the ethical committee of Tongji Medical College of HUST (Approval ID: S220).

Isolated mice colons were opened longitudinally and washed extensively with cold PBS. Then circular punches were prepared.

The punched colonic specimens were cultured in FBS-supplemented RPMI 1640 medium for 24 h. Supernatants were collected and kept frozen until ELISA assessment (25). The ELISA results were then divided by surface area (about 12.56 mm^2^) to obtain the sGRP78 and cytokines concentrations.

### 2.5 Isolation and induction of lymphocytes and Bone-marrow-derived macrophages

After extensive washing, colons were treated with digestive fluid with rapid shaking. After centrifugation, cell pellets were resuspended in 40% percoll and overlaid onto 70% percoll for centrifugation. Cells in the middle layer were collected as colonic intraepithelial lymphocytes (IELs). The remaining tissues were further digested with type IV collagenase (Roche, Basel, Switzerland) and DNase I (Roche). Lymphocytes were purified by percoll density gradient centrifugation as lamina propria lymphocytes (LPLs). Details on the isolation procedure are described in the online **Supplementary Section**.

Bone-marrow-derived macrophages (BMDMs) from mice were prepared as our previous reports (22, 31, 32). Peritoneal macrophages (PMs) were recovered from abdominal cavity lavage fluid of C57BL/6 mice, and macrophages were purified by adherence to plastic overnight (>95% F4/80^+^ macrophages). Protocols to determine macrophages polarization are provided in the online **Supplementary Section**.

### 2.6 Histologic evaluation of colitis

HE-stained colon cross-sections was scored according to four aspects: severity of inflammation (scored 0-3), extent of inflammation (scored 0-3), crypt damage (scored 0-4) and percentage involvement in the ulcer or erosion (scored 0-4). The score was determined using the following formula: overall score = “(severity of inflammation + extent of inflammation + crypt damage) × percentage involvement”. The highest score is 40.

### 2.7 Immunolabeling techniques

Immunofluorescent staining, flow cytometry analyses and western blotting were conducted on colonic tissues and in cells from spleen, mesenteric lymph nodes (MLNs), and IELs, LPLs. CBA and ELISA assays were used to determine levels of sGRP78 and cytokines in supernatants of cultured punches and in plasma. Details on the labeling procedure are described in the online **Supplementary Section**.

### 2.8 Real-time quantitative reverse transcription PCR (RT-qPCR)

Total RNA from cells was extracted and cDNA was synthesized. RT-qPCR mixtures were prepared using a SYBR Green Real-Time PCR kit (Bio-rad, Hercules, CA, USA). mRNA levels were normalized to GAPDH, and fold changes were determined using the 2^−ΔΔCt^ method. The sequences of primer pairs used are provided in the online **Supplementary Section**.

### 2.9 Protein purification

Recombinant murine GRP78 and subtilase cytotoxin subunit A (subA) were prepared as described previously (19, 33). Protein concentration was detected by the BCA Protein Assay Kit (Fdbio, Hangzhou, China). Endotoxins were removed by the Pierce™ High Capacity Endotoxin Removal Spin Columns (Thermo Fisher scientific, Rockford, IL, USA); the final endotoxin concentration in protein samples was <10 EU/mg.

### 2.10 FITC-dextran assay

All mice were fasted for 4 h and then orally administrated with 150 μl FITC-dextran (80 mg/ml). 4 h later, mice were deeply anaesthetized and the blood was collected from supra orbital sinus. After centrifugation, FITC-dextran fluorescence in serum was measure using fluorescence microplate reader with an excitation wavelength of 485 nm and an emission wavelength of 528 nm.

### 2.11 Statistical analysis

Details of data presentation, sample size (n), statistical analysis, and significance of differences are given in the figure captions. Statistical comparisons were made using GraphPad Prism 6 (GraphPad Software Inc., San Diego, CA, USA) by Student’s t test between two groups or by parametric analysis of variance (ANOVA) among multiple groups. Percentage data were analyzed by Mann-Whitney U test using SPSS 17.0 statistical software (SPSS Inc., USA). A *p*-value less than 0.05 was considered statistically significant.

## 3 Results

### 3.1 sGRP78 expression is reduced in the inflamed colon

**Figure 1:**
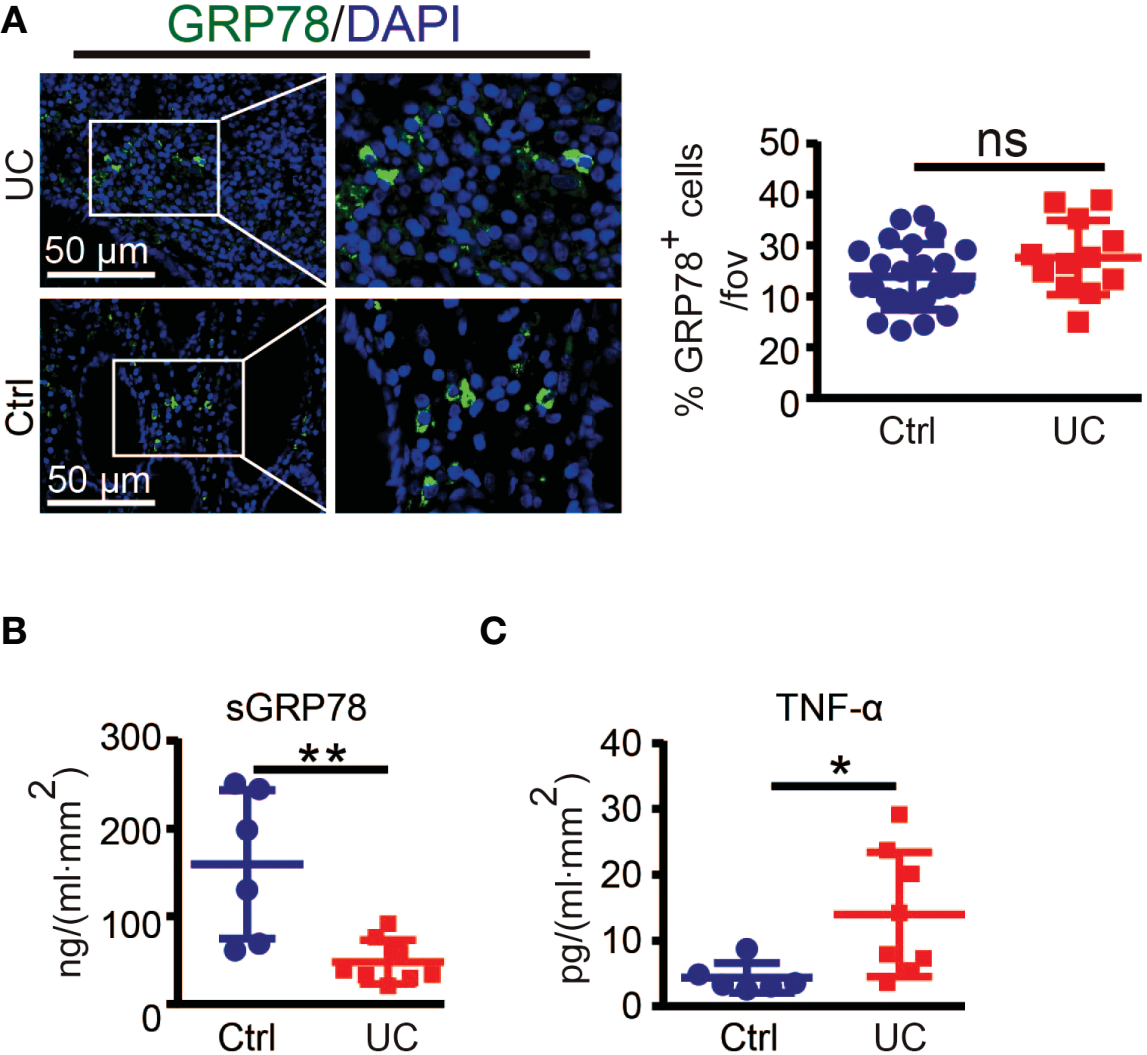
GRP78 is decreased in the intestinal mucosa of UC patients. **(A)** Representative immunofluorescent staining of GRP78 in human colonic tissues (left). Scare bar: 50 μm. The quantitative results about the percentage of GRP78 immunoreactive cells per field of view was shown in right. sGRP78 **(B)** and TNF-α **(C)** levels in the supernatants of mucosa punches collected from UC (n=8) and non-colitic control (n=6) patients. The levels were normalized to colonic surface area. Results are expressed as mean ± standard error of mean (SEM). ns, not significant. *p < 0.05, **p < 0.01.

To investigate the correlation of GRP78 with intestinal inflammation, we collected 14 human colonic specimens (8 from UC patients and 6 from colon cancer patients) to detect the expression pattern of GRP78 in the human colon mucosa. Immunofluorescent staining manifested that the frequencies of GRP78 immunoreactive cells in colitic mucosa were parallel with those in control (**Figure 1A**). However, different from immunofluorescent results, ELISA assay revealed that sGRP78 production in UC mucosa supernatants was around 47.96 ± 8.852 ng/ml.mm^2^, much lower than 159.1 ± 34.6 ng/ml.mm^2^ in non-colitic controls (*p*<0.01, **Figure 1B**). Meanwhile, TNF-α remained at higher level in UC group than in controls (**Figure 1C**).

However, there is concern that specimen near the resection margin of colon cancer patients as non-colitic control would have elevated GRP78 which would not be seen in healthy controls. Intestinal resection margins in colon cancer should be at least 5 cm between the tumor and the colonic section line. Those distal colon tissues of cancer patient maintained normal organizations (27–29). GRP78 is strongly induced in tumors and plays critical roles in the stress of oncogenesis. Although we did not detect whether GRP78 was elevated in distal colon tissues compared with cancers, in our previous investigation about hepatocellular carcinoma, it was found that compared with cancer tissues, paracancerous regions showed weak or undetectable GRP78 expression (34). Hence those distal colon tissues from cancer patients are not supposed to have elevated GRP78 and would be reliable and convinced non-colitic control.

**Figure 2:**
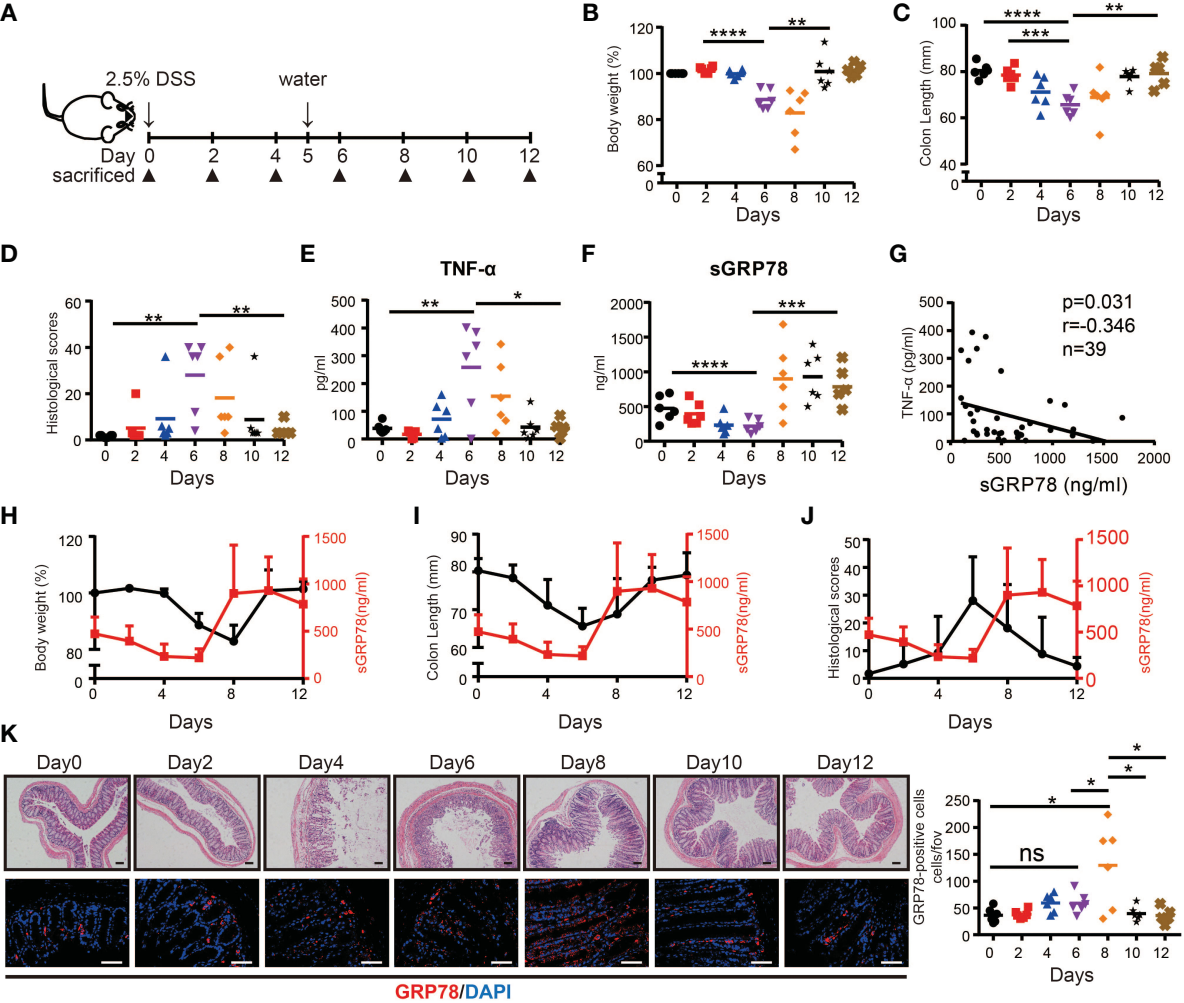
sGRP78 expression in experimental models of colitis. **(A)** Scheme of colitis induction. **(B)** Body weight was measured every two days and expressed as the percentage from day 0. At indicated time points, the colonic tissues were removed from sacrificed mice for following measurements: **(C)** Colon length. **(D)** Histological scores. Levels of TNF-α **(E)** and sGRP78 **(F)** in colonic punches supernatants. **(G)** Correlation analysis between sGRP78 and TNF-α (n=39). Pearson correlation coefficients (r) and p-values were calculated and shown. Relationship curve of sGRP78 with weight loss **(H)**, colon shortening **(I)**, and histological changes **(J)** in colitis. **(K)** Representative photomicrographs of colon sections (left) stained with HE (upper panel) or GRP78 (lower panel). (Day 0: black circle; Day2: red square; Day4: blue regular triangle; Day6: Purple inverted triangle; Day8: orange rhombus; Day10: black pentagram; Day12: brown error). Scale bar: 100 μm (upper panel) and 50 μm (lower panel). Numbers of GRP78-positive cells were calculated and shown in the right. Data represent mean ± SEM (n=5–6). ns, not significant. *p < 0.05, **p < 0.01, ***p < 0.001, ****p < 0.0001.

Above results suggested that secreted GRP78 was significantly reduced in actively inflamed human colon mucosa. Next, we tested the expression pattern of sGRP78 in DSS colitis model (**Figure 2A**) which exhibits many phenotypic features of human UC (25). Colitis was induced with 2.5% DSS in drinking water for 5 days. Consistent with the literature reports (35), mice lose 10–20% of their body weight (**Figure 2B**), associating with shortening of the colon, increasing histologic scoring and secretion of inflammatory cytokine TNF-α within active phase (**Figures 2C-E**). Within resolving and remission phase, the symptoms of colitis and above indexes recovered immediately after DSS withdrawal (**Figures 2B-E**). On this acute DSS colitis model, sGRP78 levels in colonic punches supernatants were found to gradually decline to a nadir on day 6, with marked increase above baseline by day 8 and only a slight decrease on the end of 12-day observation period (**Figure 2F**). The timing and extent of the increase were suggestive of a sGRP78’s relevance with colitis remission.

In the following correlation analyses, sGRP78 level was approved to be negatively associated with secretion of TNF-α (*p* = 0.031, r = -0.346, **Figure 2G**). It was further observed that within active phase, sGRP78 declined along with the decrease of body weight, colon length and the increase of histological score. When sGRR78 reached its nadir, disease severity indexes reached their peak values. After removal of DSS, sGRP78 level increased with the amelioration of disease severity (**Figures 2H-J**). And the rebounding of sGRP78 was 2 days earlier than that of body weight (**Figure 2H**). Immunofluorescent staining in **Figure 2K** also showed that the frequencies of GRP78-positive cells in colonic tissues fluctuated reversely with the transmural inflammation.

Collectively, these data suggest that sGRP78 is involved in the resolution of inflammatory colitis.

### 3.2 sGRP78 ameliorates DSS-induced colitis *in vivo*

**Figure 3:**
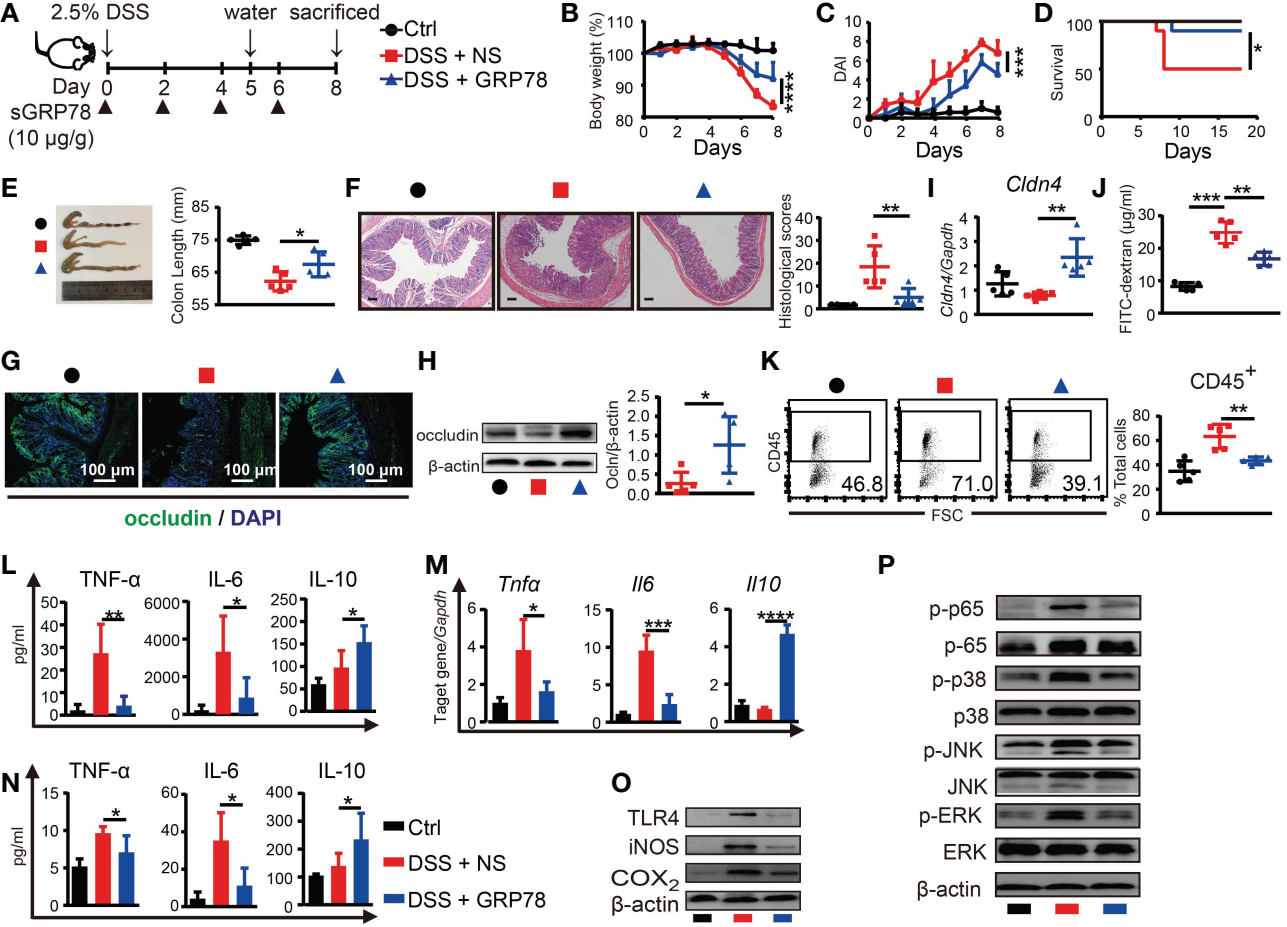
sGRP78 mitigates DSS-induced colitis and effects of sGRP78 on cytokine profile. **(A)** Timetable scheme showing DSS and sGRP78 administration in mice. Effect of sGRP78 on **(B)** weight loss, **(C)** DAI score, **(D)** survival rate (n=10) in DSS exposed mice. At the end of observation (day 8 of experiments), mice were sacrificed and the colonic tissues were removed for following measurements: **(E)** Representative gross picture of colons (left) and quantification of colon length (right). **(F)** Representative HE-staining (left) and respective colitis scores of indicated groups (right). Scale bar: 100 μm. **(G)** Representative immunofluorescence images of occludin. **(H)** Occludin and **(I)** Cldn4 levels measured by WB and RT-qPCR. **(J)** FITC-dextran assay. **(K)** Frequencies of CD45^+^ cells in single-cell suspension of colon tissues. Results are expressed as the mean ± SEM (n=5 except figure D). *p<0.05, **p<0.01. Black circle stands for vehicle control, red square for DSS+NS group and blue triangle for DSS+sGRP78 group. **(L)** Levels of cytokines in colonic tissues culture supernatant. **(M)** mRNA expression of *Tnfα*, *Il6* and *Il10* in colonic tissues. **(N)** Plasma concentrations of cytokines. IL-6 and TNF-α were measured by CBA and IL-10 by ELISA. **(O)** Western blot analysis of the expression of TLR4, iNOS, COX_2_, **(P)** MAPKs and p65 in colonic tissues. Data represent mean ± SEM (n=5). *p < 0.05, **p < 0.01, ***p < 0.001, ****p < 0.0001.

To explore whether sGRP78 supplement could effectively ameliorate colitis, recombinant mouse GRP78 was administered to DSS-treated mice as shown in **Figure 3A**. Results showed that sGRP78 significantly ameliorated DSS-induced weight loss, colonic shortening, histological inflammation, disease severity and mortality (**Figures 3B-F**), suggesting an overall improvement of intestinal inflammation.

As defective tight junction (TJ) integrity is a key pathology of colitis, in our following experiments, two main TJ components of intestinal barrier, occludin and Cldn4, were detected. Immunohistochemical staining and immunoblot manifested that expression of occludin was markedly diminished in the inflamed intestinal epithelial layer, while sGRP78 treatment maintained a relatively normal occludin pattern in the epithelium (**Figures 3G, H**). In addition, the mRNA of *Cldn4* showed the similar variation trend (**Figure 3I**). FITC-dextran assay showed that DSS-induced colitic mice had a damaged intestinal barrier with a high level of FITC-dextran exuded into serum, and sGRP78-treated mice had a low level of dextran exudation (**Figure 3J**), suggesting the restoration of TJ pattern and gut permeability by sGRP78 administration in DSS-treated mice. Such restoration of TJ integrity was accompanied by a decreased infiltration of CD45^+^ cells in sGRP78-treated colitis models (43.24% ± 1.40*% vs*. 63.32% ± 4.45% in DSS-treated mice, *p*<0.01, **Figure 3K**).

Cytokine secretion patterns are also closely associated with intestinal inflammation and IBD clinical symptoms (36). Consistent with the improvement in disease severity and colonic histopathological structure, in colitis models sGRP78 treatment significantly inhibited the production of pro-inflammatory mediators (TNF-α and IL-6) but increased the secretion of anti-inflammatory mediator IL-10 in plasma and in colonic tissues (**Figures 3L-N**). These effects were accompanied by deactivation of TLR4-MAPK signaling pathway, as indicated by the diminished phosphorylation of p38, ERK, JNK, p65 and less expressions of COX2, iNOS, and TLR4 when compared with colitis models (**Figures 3O, P**).

Above data suggested that sGRP78 administration could evidently ameliorate DSS-induced colitis *in vivo*.

### 3.3 sGRP78 induced macrophages M2 polarization in colitis

**Figure 4:**
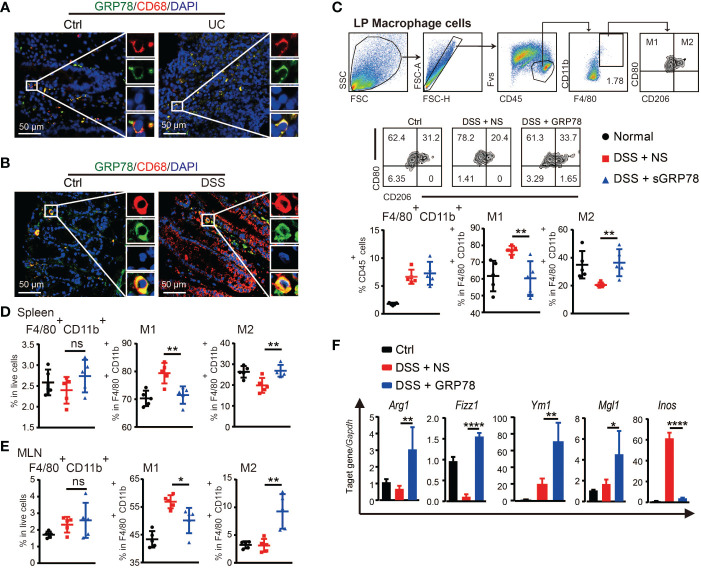
sGRP78 induced macrophage M2 polarization in colitis. Representative immunofluorescent images of GRP78 (green) and CD68 (red) stained human **(A)** and mouse **(B)** colonic mucosa. Scale bar: 50 μm. **(C)** Frequencies of F4/80^+^ cells in lamina propria (LP) and their CD80^+^ or CD206^+^ expressions. Upper: gating strategy followed to distinguish the LP macrophages. Middle: representative FCM dot plots. Lower: FCM plots based quantitative analysis for F4/80^+^CD11b^+^ macrophages. **(D)** Frequencies of indicated cell populations in spleen. **(E)** Frequencies of indicated cell populations in MLN. **(F)** mRNA expression of *Arg1*, *Fizz1*, *Ym1*, *Mgl1*, and *Inos* in mice colonic tissues (Ctrl group: black circle; DSS+NS group: red square; DSS+GRP78 group: blue regular triangle). Data represent mean ± SEM (n=5). ns, not significant. *p < 0.05, **p < 0.01, ****p < 0.0001.

Macrophages are major leukocytes in intestinal mucosa and involved in IBD through secreting pro-inflammatory cytokines under inflammatory conditions (37). Massive and persistent mucosal infiltration of macrophages are reported as one of the most evident features in ulcerative colitis (38). Our immunofluorescence results verified that in human UC and mice colitic tissues there were dense infiltration of CD68^+^ macrophages (**Figures 4A, B** and **Supplementary Figure S1**). Interestingly, a substantial proportion of these macrophages was observed to emit GRP78’s green fluorescence and the orange to yellow overlap fluorescence distributed on the membrane (**Figures 4A, B**), suggesting an action of GRP78 on colonic macrophages. As sGRP78 appeared to dampen inflammatory responses, we next examined its impact on macrophage infiltration and polarization upon DSS-induced injury. Data manifested that sGRP78 treatment did not alter the mucosal infiltration of macrophages in DSS-exposed mice, but significantly reduced CD80 expression and enhanced CD206 level on cells (**Figure 4C**). Same phenomenon was observed on splenic and mesenteric lymph node (MLN) macrophages (**Figures 4D, E**). In agreement with the upregulation of CD206 that serves as a useful marker to identify the M2 phenotype and the downregulation of CD80 that serves as a well-known marker to identify the M1 phenotype (39–42), mucosal infiltrated macrophages in sGRP78-treated colitis mice exhibited a significant increase in M2-associated genes *Arg1*, *Fizz1*, *Ym1*, and *Mgl1*, and decrease in M1-associated gene *Inos* (**Figure 4F**).

**Figure 5:**
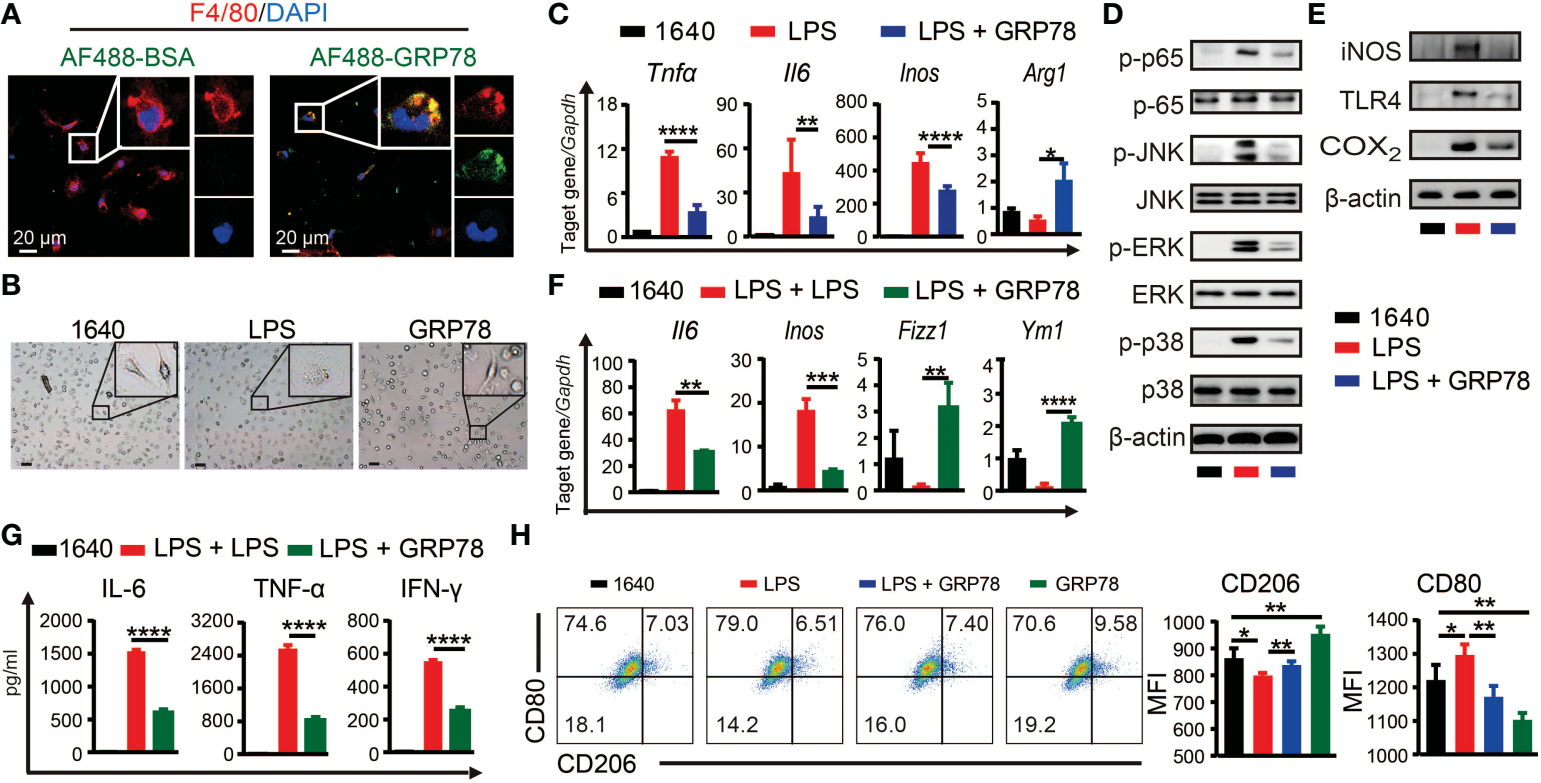
sGRP78 reverses LPS-induced macrophage M1 polarization *in vitro.* **(A)** Binding of recombinant mouse sGRP78 with F4/80^+^ BMDMs. Scale bar: 20 μm. **(B)** Representative photographs of BMDMs conditioned by sGRP78 (10 μg/ml) or LPS (100 ng/ml) for 24 h (magnification 100×). Scale bar: 100 μm. **(C)** Levels of *Arg1*, *Tnfα*, *Il6* and *Inos* in peritoneal macrophages. Western blot analysis of the expression of MAPKs, p65 **(D)**, and TLR4, iNOS, COX_2_ **(E)** of BMDMs. To further assess the effect of sGRP78 on LPS-induced macrophage, BMDMs were stimulated with LPS for 18 h and then with LPS or sGRP78 for another 12h (for mRNA detection **F**) or 24h (for cytokine release, **G**). **(H)** Levels of CD80 and CD206 on BMDMs. Error bars represent mean ± standard deviation from triplicate samples in one experiment. *p < 0.05, **p < 0.01, ***p < 0.001, ****p < 0.0001.

To investigate whether sGRP78 could directly act on macrophage to skew its polarization, BMDMs were treated with AF488 labeled recombinant mouse GRP78. Confocal imaging showed that nearly all F4/80^+^ cells could be stained with AF488-GRP78, but none with AF488-BSA (**Figure 5A**), indicating the binding of GRP78 with F4/80^+^ macrophages. Different from LPS-stimulated BMDMs exhibiting a large round appearance, sGRP78-conditioned cells exhibited an elongated spindle shape (**Figure 5B**), and dramatically upregulated their M2-associated molecules with significant downregulation of inflammatory mediators at both RNA and protein levels (**Supplementary Figure S2**), suggesting their M2 polarization. Interestingly, when sGRP78 was coadministrated with LPS, cells still downregulated their *Tnfα*, *Il-6*, *Inos*, CD80 levels and upregulated *Arg1*, CD206 levels compared with LPS-stimulated cells (**Figures 5C, H**), as well as deactivation of TLR4-MAPK signaling pathway (**Figures 5D, E**). And this trend was maintained in sGRP78-conditioned, LPS-preactivated cells (**Figures 5F, G**). These results were in line with our previous reports that GRP78-conditioned myeloid APCs still maintained the tolerogenic signature upon LPS stimulation (21).

Together, these results suggested that sGRP78 could skew macrophages towards a less inflammatory phenotype to alleviate colitis.

### 3.4 Adoptive transfer of sGRP78-conditioned BMDMs ameliorated DSS-induced colitis

**Figure 6:**
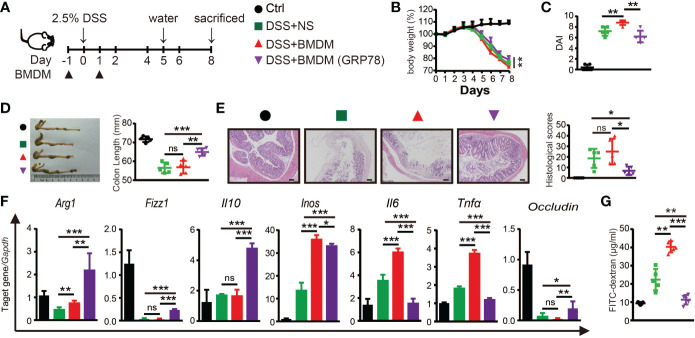
Adoptive transfer of sGRP78-conditioned BMDMs ameliorated DSS-induced colitis. **(A)** Timetable scheme showing DSS and BMDMs administration in mice. Effect of sGRP78-induced BMDMs on **(B)** weight loss, **(C)** DAI score (n=5) in DSS exposed mice. **(D)** Representative gross picture of colons (left) and quantification of colon length (right). **(E)** Representative HE-staining (left) and respective colitis scores of indicated groups (right). Scale bar: 100 μm. **(F)** mRNA expression of *Arg1, Fizz1,Il10, Inos, Il6, Tnfα, Occludin* in colonic tissues. **(G)** FITC-dextran concentrations in serum of mice. (Ctrl group: black circle; DSS+NS group: green square; DSS+BMDMs group: red regular triangle; DSS+BMDMs (GRP78) group: Purple inverted triangle). Data represent mean ± SEM (n=4-5). ns, not significant. *p < 0.05, **p < 0.01, ***p < 0.001.

The protective role of sGRP78 on DSS-induced colitis and sGRP78’s effect on M2 polarization of macrophage were proved *in vivo* and *in vitro.* To verify whether sGRP78-mediated alleviation of inflammation is due to the changes in macrophages, BMDMs were conditioned with sGRP78 and then adoptively transferred to DSS-treated mice (**Figure 6A**). Results showed that transfer of sGRP78-conditioned BMDMs significantly ameliorated DSS-induced weight loss, colonic shortening, histological inflammation, disease severity (**Figures 6B-E**) compared with colitic mice treated by control BMDMs. Consistent with the improvement in disease severity and colonic histopathological structure, transfer of sGRP78-conditioned BMDMs significantly inhibited the expression of pro-inflammatory M1 macrophage marker but increased those anti-inflammatory M2 marker in colonic tissues (**Figure 6F** and **Supplementary Figure S3**), and increased *Occludin* mRNA expression (**Figure 6F**), inhibited FITC-dextran exudation into serum (**Figure 6G**). These data suggested that GRP78 induced changes in macrophages contributed to reduction of inflammation in the gut.

### 3.5 sGRP78 selective serine proteinase subA aggravates DSS colitis

**Figure 7:**
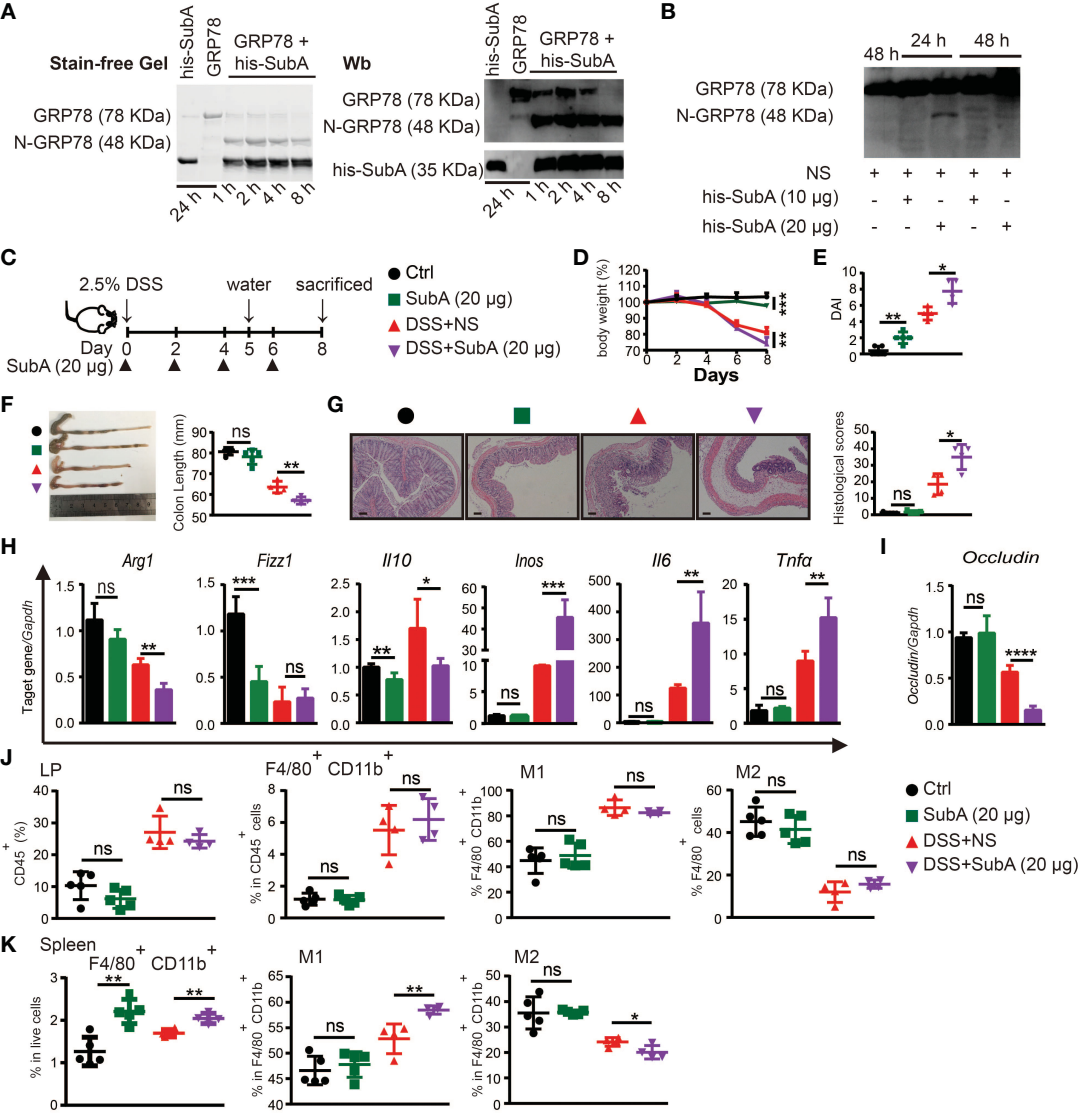
Degradation of sGRP78 aggravates DSS-induced colitis. **(A)** *In vitro* cleavage of sGRP78 (20 μg/ml) by its selective serine proteinase subA. **(B)** Cleavage of endogenous sGRP78 by intraperitoneally injected subA. **(C)** Timetable scheme showing DSS and subA administration in mice and symbols of each group (Ctrl group: black circle; subA group: green square; DSS+NS group: red regular triangle; DSS+subA group: Purple inverted triangle). **(D)** Weight loss. **(E)** DAI and **(F)** representative gross picture of colons (left) and quantification of colon length (right). **(G)** Representative HE-stained colons and respective colitis scores of indicated groups. Scale bar: 100 μm. **(H)** mRNA expression of polarization-related genes in colonic tissues. **(I)** Colonic expression of *Occludin* measured by RT-qPCR. Frequencies of CD80^+^ (M1) or CD206^+^ (M2) cells in F4/80^+^ colonic LPLs **(J)** and spleen **(K)**. Data represent mean ± SEM (n=4-5). ns, not significant. *p < 0.05, **p < 0.01, ***p < 0.001, ****p < 0.0001.

To explore whether cleavage of endogenous sGRP78 would compromise its immunoregulatory function to affect colitis self-limiting, sGRP78 selective serine proteinase subtilase cytotoxin catalytic A subunit (subA) (33, 43) was administered to DSS colitis models. **Figure 7A** showed that subA cleaved sGRP78 to produce a 48-kDa N-terminal fragment *in vitro*. And *in vivo* administered subA also caused the cleavage of colonic sGRP78 at 24h. With the prolongation of treatment time (48h), the 48-kDa fragment was further digested into pieces (**Figure 7B**).

According to above data, in the following experiments, subA was intraperitoneally injected at dose of 20 μg per mouse every other day (**Figure 7C**). As expected, subA treatment in DSS-exposed mice aggravated the colitis symptoms such as more severe weight loss, higher DAI score, more prominent shortening of the colon, and more severe histological damage (**Figures 7D-G**). In subA-exposed colitic mice, for macrophage polarization-associated genes, M2-related *Arg1* and *Il10* were significantly downregulated in the colonic tissues while with a significant upregulation of M1-related *Inos*, *Il6* and *Tnfα* (**Figure 7H**), *occludin* expression was conspicuously decreased (**Figure 7I**), and CD80 upregulation and CD206 downregulation on splenic macrophages were also observed (**Figures 7J, K**). These results indicated that selective inactivation of sGRP78 by subA could aggravate DSS-induced colitis, thus further demonstrating the role of sGRP78 in relieving intestinal inflammation.

Interestingly, subA exposure to healthy mice also caused intestinal inflammation and colitis symptoms to some degree (**Figures 7D-K**) by specific cleavage of endogenous sGRP78, implying that physiologically secreted GRP78 participates in the maintenance of gut immunological homeostasis.

## 4 Discussion

In response to stresses, intestinal cells mobilize some endogenous HSPs to confront injury (6). For instance, oxidative stress induced colonic mucosa or cultured mucosal epithelial cells to upregulate heat shock proteins (HSP) 25 to confer cytoprotection (44). Dietary components, food additives and physical activity have a strong impact on HSP70 induction to protect intestinal epithelium (8). Despite the emerging evidence supporting an important role for sGRP78 (HSP70 family) in inflammation resolution, studies on its role in colitis resolution are incomplete. Gut is an important site of immune tolerance with the constant assault of food antigens and its billions of resident microbes (45). This study unraveled that, in line with the milieu of intestinal tolerance, sGRP78 was spontaneously produced to a high level in gut, as well as in liver (24), another site of immune tolerance, inferring that this degree of sGRP78 production probably contributes to the maintenance of gut immune homeostasis. This hypothesis was partly proved by the fact that administration of sGRP78 selective serine proteinase subA could induce mild intestinal inflammation. In the following studies, it was found that, upon colonic injury, sGRP78 was significantly reduced in colitic mucosa of UC patients and of acute DSS colitis model. Interestingly, once colonic injury was removed, in experimental colitis models sGRP78 level rebounded quickly and lastingly with subsequent weight restoration and amelioration of disease severity. Correlation analyses confirmed the negative association between sGRP78 and the most important cytokine that mediates intestinal tract inflammation, TNF-α (46). All these results suggest that sGRP78 acts as a naturally occurring regulator orchestrated to prevent excessive inflammation and its downregulation is involved in the defective immune tolerance in the gut. It needs our further study to investigate the cellular origins of sGRP78 and the causal relationship between ulcerative colitis and the fluctuation of sGRP78.

The treatment goal in ulcerative colitis is the induction and maintenance of remission. The primary drugs used in UC include 5-aminosalicylic acid, steroids, azathioprine, 6-mercaptopurine, tofacitinib (47, 48) and some biologic agents, including anti-TNF (infliximab, adalimumab, certolizumab, and golimumab), anti-integrins (vedolizumab, natalizumab) and anti-IL12/23 agent (ustekinumab) (49). Despite the number of available medications, there are appreciable rates of primary non-response, loss of response, or adverse reactions (50) thereby necessitating additional treatment options. As a non-specific internal factor produced by gut for the maintenance of homeostasis, sGRP78 has potential to be a better option. Here, we provide evidence that administration of sGRP78, which is evidently downregulated in both experimental and human IBD, protects against inflammation in the DSS colitis model in a preventive manner. And specific cleavage of endogenous sGRP78 by its selective serine proteinase subA could aggravate DSS colitis. On the basis of our results, sGRP78 may represent a novel therapeutic approach for active UC.

When exploring the underlying mechanism, it was found that sGRP78’s effect was involved in activity of macrophages. This was in accordance with our previous reports that sGRP78 could bind with BMDMs (22) and hepatic macrophages (24) to exert its immunoregulatory functions. Macrophages, an important component of the innate immune response, are also a key regulator of intestinal microenvironment homeostasis (38, 51). They weigh more on the UC pathological progression than neutrophils (52). Macrophages maintain intestinal immunological homeostasis *via* balancing the expression of many pro\anti-inflammatory cytokines (36), induction or expansion of regulatory T cells in the intestine (53), repairing mucosal layer to restore barrier integrity (54), and so on. In our research, consistent with the improvement of disease severity indexes and colonic histopathological structure, sGRP78 was found to promote macrophage polarization toward the M2-like phenotype, reverses the inductive effect of LPS on macrophage M1 polarization to subsequently downregulate proinflammatory cytokine production while upregulate the secretion of IL-10. And transfer of sGRP78-conditioned BMDMs also reduced inflammation in the gut. Meanwhile, sGRP78 administration normalized the expression of tight junction proteins (occludin and claudin-4) to prevent the infiltration of immune cells in inflamed tissue, another hallmark of IBD (55), thus facilitating remission of acute colitis. Models for chronic DSS colitis and colitis-dependent neoplasia are under developing in our lab to gain more mechanistic insights into sGRP78 on adaptive immunity.

We previously reported that it is through downregulation of TLR4 on myeloid cells that sGRP78 favors the resolution of inflammation (22). In this research, sGRP78 was also found to impair the expression of TLR4 and TLR4-dependent MAP-kinases pathway and NF-κB pathway, which were proved to contribute to the regulation of macrophage polarization in UC and cytokine production (56). Such upstream interaction leads to the downstream decrease of all the sequel of events triggered by the TLR4 activation, including the decrease of p38-pERK-pJNK expression and phosphorylated p65 subunits expression.

In summary, although further studies are required to better define the sGRP78 anti-inflammatory effects in UC onset/progression, we propose sGRP78 as a new drug able to control the acute phase of intestinal inflammation occurring in UC, profoundly and beneficially impacting on macrophage polarization and, mainly *via* inhibiting the TLR4-dependent MAP-kinases and NF-κB pathways. As a naturally occurring regulator, sGRP78 might be regarded as a potential, innovative, less toxic tool for UC treatment.

## Data availability statement

The raw data supporting the conclusions of this article will be made available by the authors, without undue reservation.

## Ethics statement

The studies involving human participants were reviewed and approved by the ethical committee of Tongji Medical College of HUST (Approval ID: S220). The patients/participants provided their written informed consent to participate in this study. The animal study was reviewed and approved by The Ethics Committee of Tongji Medical College of Huazhong University of Science and Technology (HUST, Approval ID: S1161/S2534).

## Author contributions

LZhao, YL and PL proposed the conception and design of this study. LZhao, YL, ZG, XZ, HL, YG, TL, LT, LZhu executed related experiments and acquisition of data, or analysis and interpretation of data. LZhao, YL and PL drafted the article or revised it critically for important intellectual content. PL, YH, GS, JT finally approved the version to be submitted. All authors contributed to the article and approved the submitted version.
