# Supplementary material for: Secreted glucose regulated protein78 ameliorates DSS-induced mouse colitis
Source: Front Immunol. 2023 Jan 26;14:986175. doi: 10.3389/fimmu.2023.986175 (PMC9909966; doi:10.3389/fimmu.2023.986175)

**Supplementary** **materials and methods**

**Disease activity index (DAI) in mice**

The DAI scale is based on the evaluation of different parameters characterizing experimental colitis induction and progression. Mice body weight, stool consistency and rectal bleeding were monitored daily (from day 0 to 8) by an observer blinded to the treatment. The DAI was scored according to the following three parameters: weight loss (0 = none; 1 = 1 to 5%; 2=6 to 10%; 3 = 11 to 15%; 4 = >15%); stool consistency (0 = normal; 1 = soft but still formed; 2 = soft; 3 = very soft, wet; 4 = watery diarrhea) and fecal occult blood (0 = occult blood test-negative, 1 = occult blood test-positive, 2 = slight, 3 = blood traces in stool, 4 = gross rectal bleeding.).

**Isolation of intestinal lymphocytes**

After extensive washing, colons were treated with digestive fluid (RPMI 1640 medium containing 3 mM EDTA and 1 mM DTT) with rapid shaking. After centrifugation, cell pellets were resuspended in 40% percoll and overlaid onto 70% percoll for centrifugation. Cells in the middle layer were collected as colonic intraepithelial lymphocytes (IELs). The remaining tissues were further digested for 1 h with 1 mg/ml type IV (Roche, Basel, Switzerland) collagenase and 200 μg/ml DNase I (Roche) in RPMI 1640 medium. The suspension was separated from undigested tissue by filtration through 200-mesh sieves and lymphocytes were purified by percoll density gradient centrifugation as lamina propria lymphocytes (LPLs).

**Immunofluorescence and flow cytometry analyses**

For immunofluorescence staining, anti-GRP78, anti-CD68 and anti-occludin (all from Abcam, Cambridge, UK) were used as the primary antibody. Detection was performed with corresponding fluorescent-conjugated secondary antibodies. Nuclei were stained with DAPI (Beyotime, Beijing, China). Images were captured using a fluorescence microscope (Olympus, Tokyo, Japan).

Cells from spleen, mesenteric lymph nodes (MLNs), IELs, and LPLs were blocked with anti-CD16/32 and then were stained with anti-FVS (V450), anti-F4/80 (PE), anti-CD11b (APC-CY7), anti-CD80 (percp-cy5.5), anti-CD86 (APC), anti-CD45 (BV510) and anti-CD206 (FITC). Cells were tested by BD Verse flow cytometer (BD Biosciences). Data were analyzed using FlowJo software (FlowJo).

Primary BMDMs were blocked with anti-CD16/32 and then were stained with anti-FVS (V500), anti-F4/80 (BV421), anti-CD11b (PE-CY7), anti-CD80 (PE), anti-CD206 (APC). Cells were tested by BD Verse flow cytometer (BD Biosciences). Data were analyzed using FlowJo software (FlowJo).

**Cytokine measurements**

In supernatants of cultured punches and in plasma, sGRP78 (Enzo Biosciences, Lausen, Switzerland) and IL-10 (Biolegend, San Diego, USA) levels were detected by ELISA, TNF-α, IL-6 and IFN-γ by cytometric bead array (CBA, BD Biosciences, San Jose CA, USA) according to the manufacturer’s protocol. Data acquisition for CBA experiments was performed with the FCAP Array software (BD). sGRP78 levels in cultured punches supernatant were normalized to colonic surface area to determine the capacity of per unit area mucosa to secrete GRP78.

**Western blotting**

Mouse colonic tissues were homogenized in RIPA lysis buffer (Beyotime) to obtain cytosolic extracts. Extracts were resolved by SDS-PAGE gels and transferred onto PVDF membranes (Millipore, Molsheim, France) that were saturated with 5% skim milk and then probed with primary antibodies. Membranes were then incubated with specific secondary antibody conjugated to horseradish peroxide. Immune complexes were revealed by ECL detection system (Clinx Science Instruments, Shanghai, China). The following antibodies were used: β-actin and occludin (Proteintech, Wuhan, China), COX_2_, p-p38, p38, p-JNK, JNK, p-ERK, ERK, p-p65, p65 (all from Cell Signaling Technology, Beverly, MA, USA), TLR4, iNOS (Santa Cruz Biotechnology, Santa Cruz, CA, USA). Pixel density was analyzed by Image J software (NIH). β-actin was used as a loading control.

**Real-time quantitative reverse transcription PCR (RT-qPCR)**

Primer pairs used were as follows: 5'-GCTGAGTATGTCGTCCAGT-3' and 5'-GTTCACACCCATCACAAAC-3'(*Gapdh)*; 5'-TCAGTTCTATGGCCCAGACC-3' and 5'-CTTTGAGATCCATGCCGTTGG-3'(*Tnf-α*); 5'-CGGAGAGGAGACTTCACAGAG-3' and 5'-ATTTCCACGATTTCCCAGAG-3'(*Il-6*); 5'-GCTGCCAGGGTCACAACTT-3' and 5'-AACAGCTCAGTCCCTTCACC-3' (*iNos*); 5'-CCTTGGCTAACAGTTTTGCCTT-3' and 5'-AACAGCTCAGTCCCTTCACC-3'(*Ym1*); 5'-CAAGACTATGAACAGATGGGCCT-3' and 5'-AGGAGATTGATGGGAGA GGACA-3' (*Fizz1*); 5'-TGGCCTGAAGCTGACAAGTA-3' and 5'-AGGC CGATCCAACTAACCACAT-3'(*Mgl1*); 5'-CAGGACTTTAAGGGTTACTTGGGT-3' and 5'-GCCTGGGGCATCACTTCTAC-3' (*Il-10*); 5'-CCACTCTGTCCACATTGCCT-3' and 5'-CTTTGCACAGTCCGGGTTTG-3' (*Cldn4*); 5'-CCCCTCTTTCCTTAGGCGAC-3' and 5'-TTCAAAAGGCCTCACGGACA-3' (*Occludin*).

**Polarization of macrophages**

To test the effect of sGRP78 on macrophages polarization, cells (1×10^6^) were treated with sGRP78 (10 μg/ml) or LPS (100 ng/ml, Sigma). The mRNA and protein levels of specific markers (*Arg1*, *Fizz1*, *Ym1*, *Mgl1*) and cytokines (TNF-α, iNOS, IL-6 and IL-10) were measured at 4h and 24h timepoints, respectively. For assessing the plasticity of macrophages, cells were preactivated with LPS for 18 h, followed by thoroughly washing and stimulation with sGRP78 or LPS for an additional 12 h or 24 h before phenotyping.

**Figure S1: The number of CD68 positive cells in human colonic tissues per visual field is shown below.**


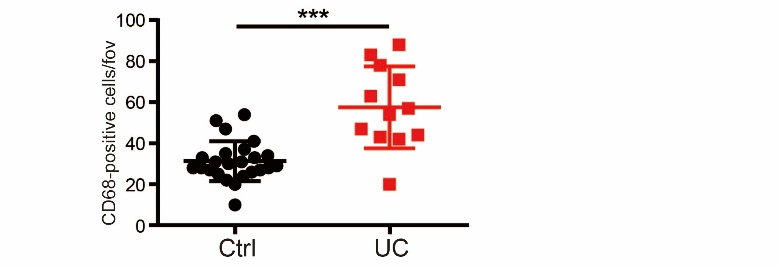


**Figure S2: sGRP78-conditioned cells upregulated their M2-associated molecules with significant downregulation of inflammatory mediators at both RNA and protein levels**

**
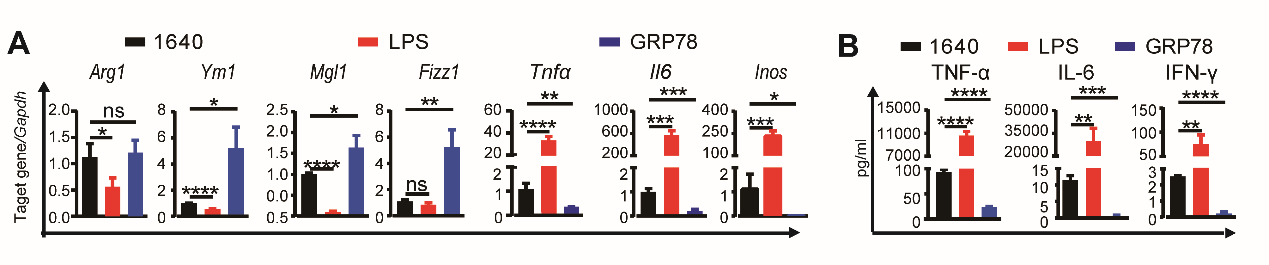
**

1. mRNA expression of polarization-related genes in BMDMs, 4h after conditioning.
2. Cytokine release in BMDMs, 24h after conditioning.

**Figure S3: sGRP78-BMDMs treatment decreased IL-6 (left) and TNF-α (right) secretion in serum.**


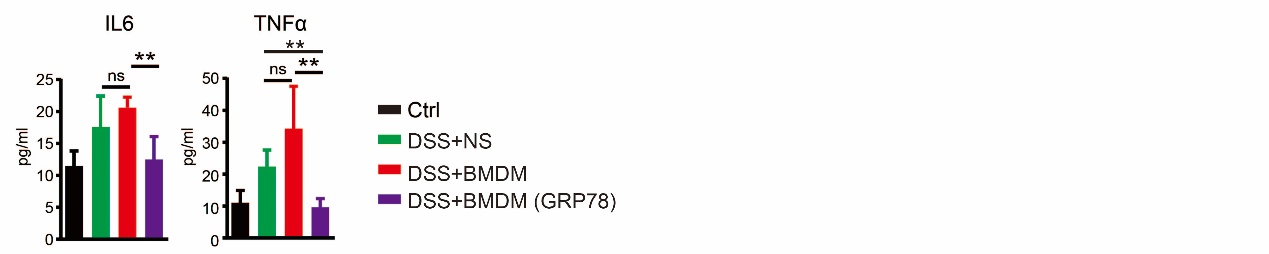

Supplement: Supplementary file 1 [file DataSheet_1.docx]
